# Placement of Optical Sensors in 3D Terrain Using a Bacterial Evolutionary Algorithm

**DOI:** 10.3390/s22031161

**Published:** 2022-02-03

**Authors:** Szilárd Kovács, Balázs Bolemányi, János Botzheim

**Affiliations:** 1 Department of Mechatronics Optics and Mechanical Engineering Informatics, Faculty of Mechanical Engineering, Budapest University of Technology and Economics, 4-6 Bertalan Lajos Street, 1111 Budapest, Hungary; bolemanyibalazs@edu.bme.hu; 2 Department of Artificial Intelligence, Faculty of Informatics, Eötvös Loránd University, Pázmány P. Sétány 1/A, 1117 Budapest, Hungary; botzheim@inf.elte.hu

**Keywords:** sensor placement, route detection, evolutionary computing

## Abstract

This paper proposes an optimization framework for terrain large scale optical sensor placement to improve border protection. Compared to the often used, maximal coverage of an area approach, this method minimizes the undetected passages in the monitored area. Border protection is one of the most critical areas for sensor placement. Unlike traditional border protection solutions, we do not optimize for 2D but for 3D to prevent transit. Additionally, we consider both natural and built environmental coverings. The applied environmental model creates a highly inhomogeneous sensing area for sensors instead of the previously used homogeneous one. The detection of each sensor was provided by a line-of-sight model supplemented with inhomogeneous probabilities. The optimization was performed using a bacterial evolutionary algorithm. In addition to maximizing detection, minimizing the number of the applied sensors played a crucial role in design. These two cost components are built on each other hierarchically. The developed simulation framework based on ray tracing provided an excellent opportunity to optimize large areas. The presented simulation results prove the efficiency of this method. The results were evaluated by testing on a large number of intruders. Using sensors with different quantities and layouts in the tested 1×1×1 km environment, we reduced the probability of undetected intrusion to below 0.1% and increased the probability of acceptable classification to 99%.

## 1. Introduction

This study aims at an optimization framework for terrain large scale optical sensor placement. Appropriate sensors are crucial for the operation of any automated system. The right number and position of sensors are essential for a sensor system’s efficient and reliable operation. The camera is one of the essential sensors in terms of surveillance. It was chosen for this study since it is reasonably priced and provides an information-rich and easy-to-understand signal. It is also crucial to consider the environment in which sensors are placed. The uniqueness of this article is not to cover the entire area but to detect any intruders. It is not necessary to cover all points of the area for detection, but it is enough to find the targets at one point in their route.

The real-life importance of this topic is protecting critical areas such as power plants, military facilities, and country borders. To be more efficient, it is high time to reconsider the previously used 2D models. Hence, these models focused on people, objects located on the ground, and land vehicles. In the case of flying targets, the 3D environment study is inevitable. Due to their relatively easy availability, flying targets such as drones or kites are more common as potential intruders. They can both be used for smuggling goods and gathering confidential information [1,2,3]. Equipped with a camera, they can be sent as an outpost to avoid the border patrols and find the proper timeframe for crosses without being caught. In addition to drone threat, summary studies were conducted on the applied sensors for drone monitoring and countermeasures against drones [3]. In line with the European Horizon projects [4], border protection is a priority for future years. One possible way to protect borders is through aerial reconnaissance. Long-term aerial reconnaissance can be achieved using airships and solar panels. Wind loading and easy visibility are the main disadvantages of airships, but their operation in a multimodal system can provide significant advantages. Sensor placement plays an important role in several other areas. Well-equipped sensor nodes with different sensors to monitor the environment and structure can be effectively used for social distancing and emergency management in a sensor network. Such a system has been tested in a park to manage visitors and create a favorable route for them to avoid crowding and plan an escape route in the event of an emergency [5].

This article has developed a new simulation-based approach to border management. The simulation uses ray tracing for effective application in highly inhomogeneous environments. Optimization was performed in 3D to prevent undetected ground and air crossings.

In Section 2, topic-related literature is reviewed. In Section 3, the simulation model is detailed. Section 4 describes the optimization method. Experimental results are presented in Section 5. Lastly, the conclusions and further works are summarized.

## 2. Related Literature

There are several sub-areas of border management, the two largest being physical border protection and psychological border management. The second’s purpose is the study of people’s behavior and psychological profiling [6]. The first is for the detection, localization, and prevention of illegal border crossings. In addition to high-altitude air traffic and ground crossings, the threat posed by low-altitude drones has increased over the last decade. The literature review can be divided into four main parts. The first part presents the sensors applicable to the boundary and area protection task and the related drone detection literature. The second topic is dynamic border protection methods. The third topic is static sensor placement. The last part contains sensor placement methods for more specialized tasks. Several studies have addressed the surveillance of drones [7]. The applied sensors for drone surveillance: camera (mono, RGB [8,9,10,11,12,13,14], multi-, hyperspectral, short-/longwave infrared [14]), radar [15], radio direction finder [16,17,18], acoustic (single [14,19,20], array, matrix) and laser detection and ranging. Various fusion techniques [14,21,22] have also appeared, mainly with visual, infrared, and acoustic sensors. In the case of ground transit, the use of geophones is also common [23,24]. There are static, dynamic solutions for boundary surveillance. Dynamic solutions include different patrol mechanisms [25,26]. Generally, short—a few hours—flight times are typical for drones [27], but developments aiming at flight times of several hours [28] also exist.

The border surveillance is often concentrated only on ground intruders, so 1D line arrangements are common in theoretical sensor placement methods. In most cases, sensors had uniform disk-shaped sensibility decreasing towards the edges [29,30,31,32,33]. Radars are well suited for area protection due to their large field of view [31]. Two dimensional (2D) coverage studies are more common in the literature than articles on border protection. Akbarzadeh et al. investigated an optimal sensor coverage in 3D elevation terrain and the built environment. The optimal 2D coordinates and horizontal and vertical angular positions of each sensor were optimized by simulated annealing, the Limited-memory Broyden–Fletcher–Goldfarb–Shanno algorithm, and the Covariance matrix adaptation evolution strategy [34]. Unlike in the natural homogenous non-flat environments in the built-in environment, the sensor placing goal was to cover inhomogeneous flat surfaces. Altahir et al. developed a weighted coverage model for installing camera surveillance systems. The placement was based on a 2D risk map in 3D space. Inversely, the sensors were placed based on 2D weighted coverage demand [35]. As a continuation of their work, they used dynamic programming as a discrete optimization for 2D generated urban layout [36]. Various aspects, such as power supply, energy efficiency, sensor lifetime, reliability, greedy coverage and the placement of the controllers in the sensor network, can be considered for the sensors’ placement [32,37,38]. An obvious solution for a wireless sensor network is to use renewable energy with a rechargeable battery [32]. The energy efficiency can also be maintained by the timing of the sensors [39]. The goal is to ensure adequate coverage even in the event of outages. In the case of a fail, relocating nodes can provide an excellent solution to hold the uniformity of 2D coverage [40]. Energy supply is also an important aspect of border surveillance. Dong et al. implemented a boundary monitoring procedure with solar-powered sensors. In addition to the surface coverage, time optimization was also applied due to the limited energy of the sensor’s battery [29,30]. The method was later expanded to include adaptive sensing range adjustment for energy-efficient, time-aligned alignment of sensors [41]. Another aspect of sensor placement is localization, which requires signal strength, time, or direction data from different sensors. Xu et al. investigated ideal sensor placement for single target localization based on circular time of arrival. The optimality criterion is to minimize the trace of the inverse Fisher information matrix [42]. Xu’s hybrid localization procedure study is for single static target localization using the hybrid received-signal-strength, angle-of-arrival, and time-of-arrival measurements on the 2D plane [43]. Akbarzadeh et al. examined a new optimization approach for temporal coverage. The essence of temporal coverage is to cover the area around the most probable position of the target point with the available sensors. It was concluded that individual control of each sensor in series works better all at once [44]. After detection, target tracking and localization is the next important task [45,46,47]. Another exciting research area is the replacement of a temporarily failed sensor for localization. Pedrollo et al. trained a neural network to be a virtual sensor, replace unavailable sensors, and generate synthetic but still realistic data [48]. Another similar task is to observe 3D objects. De Rainville et al. created a framework for mobile robotic sensor placement with covariance matrix adaptation evolution strategy optimization. The mobile robots were equipped with optical sensors. The optimization goal was maximization of the pixel density on the area [49]. Herguedas et al. examined the optimal sensor placement for deformable bodies [50]. The procedure was later improved using RGB-D cameras [51]. An important area of the Optimal Sensor Placement Problem is the vibration measurements in various structures such as bridges [52]. The problem examines small dimensionally discrete sensor placement. Zhang et al. examined the coverage-based optimization of different bodies with different evolutionary algorithms [53]. Spielberg et al. performed a sensor placement task during the soft robotics simulation to monitor the inside of the soft robot [54].

## 3. Modeling

Simulation-based optimizations are becoming more common. Creating a suitable simulation environment has a competitive speed compared to a complex analytical solution. The studies in the simulation are very flexible and illustrative. In the field of sensor placement, simulation-based solutions are less common and are not applied for border protection. Nevertheless, the simulation-based approach has many advantages compared to the traditional analytical methods. Simulation is much more flexible, making it easy to examine even dynamic environments, and is easier to expand and apply for new tasks.

During the simulation, the signal’s path between the object and the sensor was emphasized. Reflections were not considered during ray tracing because, in the studied natural environment and the use of optical sensors, this is not significant. Absorption and transparency were calculated for signal propagation. Algorithm 1 contains the process of applied signal propagation.
**Algorithm 1** Ray Tracing**function**Ray Tracing(Sensors, Intruders, Environment)    **for** $Intruders^{\prime }Route$ **do**        **function** Field of view check(Sensors, Intruders)           **return** true/false, Ray strengths, Distance and Angular differences        **if** Intruders in the sensors’ field of view **then**           **function** Ground crossing and background(Positions, Ground)               **return** true/false, Closest ground backgrounds for sensors and intruders           **if** Signal not cross the ground **then**               **function** Built crossing and background(Positions,Built elements)                   **return** true/false, Closest Built element backgrounds for sensors and intruders               **if** Signal not cross built elements **then**                   **function** Vegetation crossing and background(Positions, Vegetation, Ray strength)                       **return** Ray strengths, Closest Vegetation backgrounds for sensors and intruders                   **if** Signal greater then the cut value **then**                       **function** Cloud crossing and background(Positions, Clouds, Ray strength)                          **return** Ray strengths, Closest Cloud backgrounds for sensors and intruders           **function** Select closes background(backgrounds)               **return** Closest background    **return** Ray strengths, Backgrounds, Distance and Angular differences

The disadvantages of simulation are that it approximates reality. Simulation neglections tend to produce better results than reality, so neglections must be considered in evaluating the results. The main difficulties in object detection are the visibility of the object, the background, the weather conditions, and the influence of the sun. In this article, the visibility and background attenuation of the object has been taken into account. Weather can significantly degrade detection for some sensors [55,56]. Three different effects can occur: reduction of visibility, particles appearing in the image, and, in the case of optical sensors, particles on the detector can obscure or blur regions. The first is the inevitable decrease in most optical sensors’ detection, which can be considered as distance decreasing [55]. Particles can be filtered [57]. It is difficult to consider particles’ blur and covering effect. One possibility for optimization is to punish the high angular position of the sensors. Another solution is simulating weather conditions [58]. The sun degrades the detection to different degrees depending on the quality of the optics. The sun’s position can be calculated according to its geographic location [59]. In most cases, only the distance from the sensor is used for goodness calculation. In the prepared simulation, the ray passaged through the medium in the environment weakens the signal further. In addition to the strength of the signal, the signal-to-noise ratio from a detection perspective is more important. No model has been developed for optical sensors to test the signal-to-noise ratio for detection. This area is the best developed in the case of radar [60]. Signal-to-noise ratio based detection can be divided into four main parts: transmitter noise, receiver noise, the signal of the object to be observed, and background noise. For optical sensors in the field, the transmitter noise can be considered as the slow variation of sunlight. The receiver noise is mostly considered as the optical signal-to-noise ratio, containing dark current noise and spatial frequency transmission of optics. The signal is the visibility of the object. Background noise can be taken into account by the background environment. In this paper, the detection model has been compiled with the background in addition to object visibility, and the transmitter and receiver noise is not discussed. The receiver noise is sensor-specific and can be added to the discrete properties of the sensors. Based on the implemented ray tracing, the transmitter noise can be estimated by adding sun and other light sources. Based on the type of background, the estimated detection probability decreased. Table 1 shows the effect of background on the signal strength.

### 3.1. Environment Model

**Figure 1:**
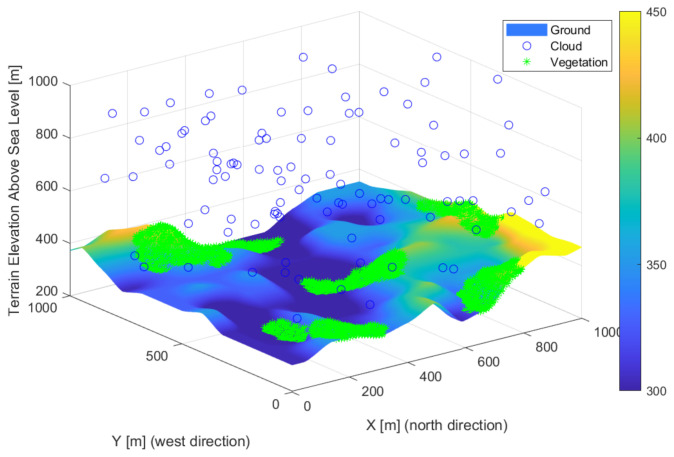
Model of the environment used for the studies, with elevation map, vegetation, clouds.

Terrain, clouds, vegetation and artificial built elements formed the modeled environment. Accurate elevation data were loaded during the design of the environment. The vegetation was loaded from a random vegetation map. The clouds and buildings were randomized. Automated loading of the entire environment is not yet implemented. The elements of the environment have different properties that are required for the placement of optical sensors. Clouds were shaped as orbs with position, size, absorption, and transmission properties. The vegetation was modeled with orbs shapes with position, size, absorption, and transparency. The constructed elements were considered rectangles with the property of position, orientation, and size. The simple shapes allow a quick parallel calculation, and more complex elements can be built. Figure 1 shows how the examined elements can construct the environment. A random vegetation map was added to the real elevation map. Clouds were generated at random locations and transparency in each iteration. Changing environmental elements increase robustness and are more realistic.

### 3.2. Sensor Model

**Figure 2:**
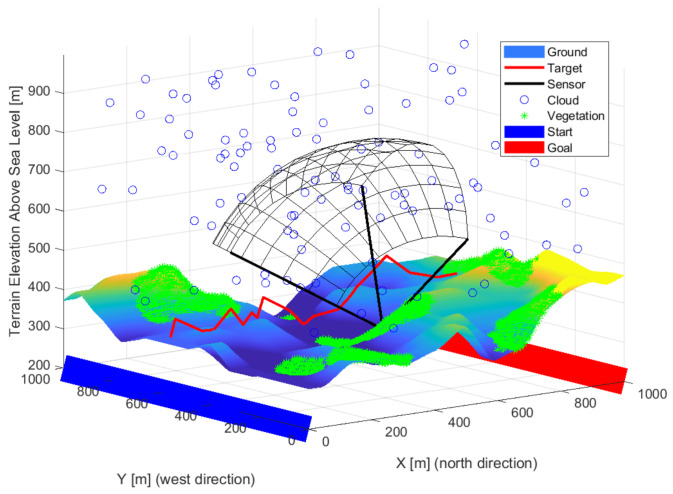
The detection field of a sensor is plotted black. The red line exemplifies a possible route of an object.

Pinhole camera models were used with focus distance. The field of view and distance were calculated based on the focal length and sensor-specific typical detector size and resolutions. The object’s size and its minimal pixels representation have an essential role in calculating visibility distance. Maximal signal strength is considered half of the visibility distance, and then it decreases linearly. The value calculated from the distance gave the initial value of the signal propagation. A crop value can be set for the detectable signal minima. The sensors have a position, orientation, and focal length properties for optimization. The detection range of a sensor plotted black is shown in Figure 2 illustrating the optimization result when only one sensor is used. A red line indicates a possible intruder route. The maximum visibility distance was determined during the optimization up to two-pixel imaging of a one-meter target. The real visibility is much smaller visibility due to the environment and background. The four-pixel projections are plotted in figures, which is better related to the visibility. A larger pixel representation is recommended for a basic classification.

### 3.3. Target Model

**Figure 3:**
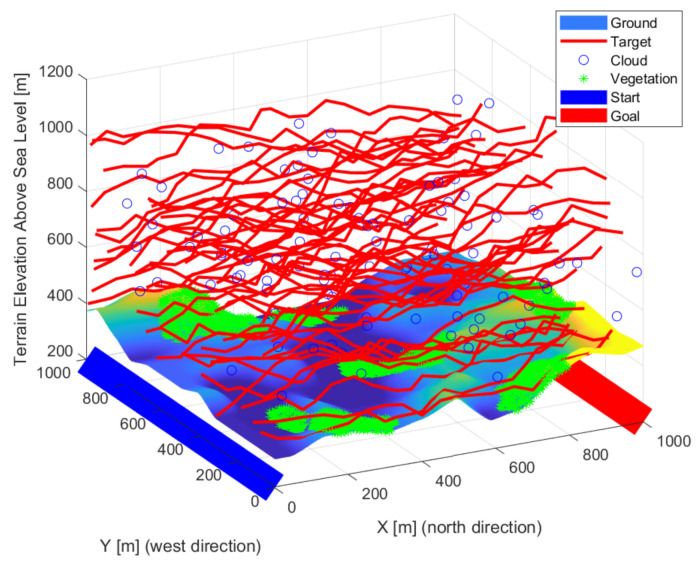
New routes (red lines) were generated at each iteration step.

**Figure 4:**
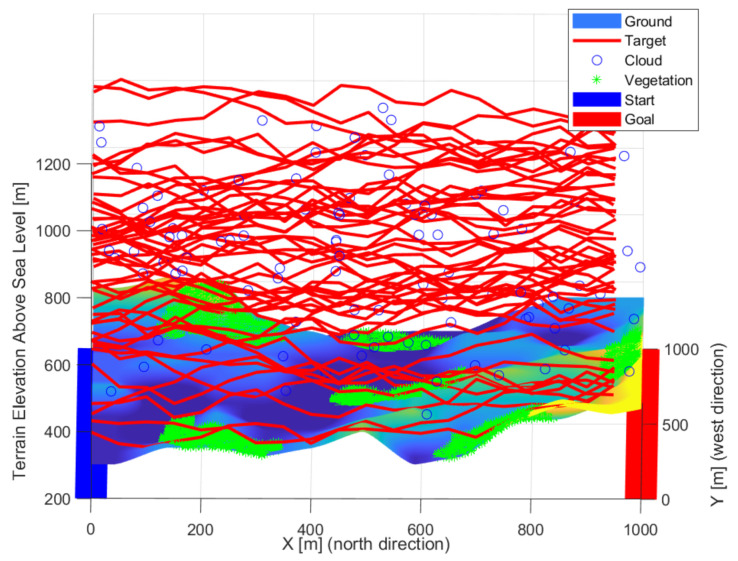
The different paths (red lines) of the objects quasi evenly filled the study area.

**Figure 5:**
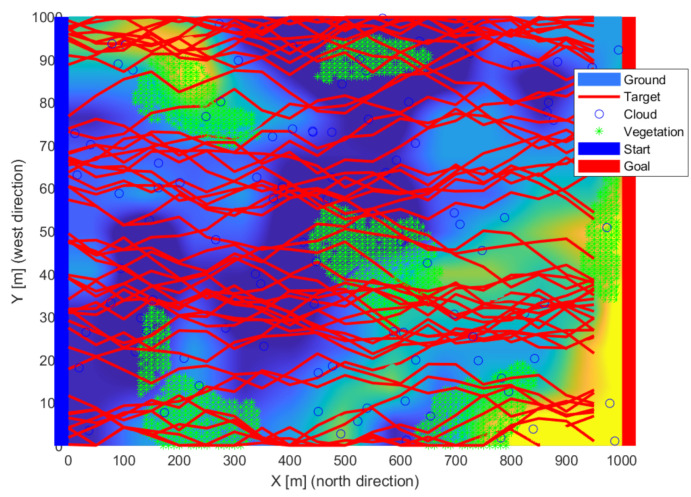
Some of the routes were complex despite the simple generation.

Flying objects were defined in the simulation, so it was necessary to examine the 3D space instead of the usual 2D. These objects move from one edge of the simulation space (x = 0) to the other (x = xmax). Any point above the surface of the starting plane of the space (x = 0) was a possible starting point. During the route planning, the area was divided evenly along the transit direction. For the other coordinates, the maximum deflection from the previous position was calculated based on the object’s top speed. The object moved randomly above the surface, between the maximum deflection and the straight direction. Objects have a size, minimal pixel representation, initial position, maximum speed, and a random path between the two edges of the study range properties. Based on the initial properties, a random path was calculated and added to the properties. Straight diagonal and mixed routes were generated in each iteration, a sample is plotted in Figure 3, Figure 4 and Figure 5. The maximum observability is sought on each route’s evaluation. The different paths (red lines) of the objects quasi evenly filled the study area. Due to the gaps, previous observations were also weighted, giving momentum to the optimization and smoothing the cost function. Some of the routes were complex despite the simple generation. Lateral, curved movements have also appeared, in addition to straight and near-straight passing attempts. Using multiple intruders in simulations with random patches results in some complex patches. The cost function considers the undetected paths with greater weight, so optimization better secures “quasi” intelligent routes. An excellent way to reduce the number of simulations is to use smarter intruders. Pinball and flood-fill algorithms can be a good solution for intelligent intruders [61].

## 4. Optimization

### 4.1. Objective

The goal is to prevent unnoticed passage with minimal sensor use. It is necessary to estimate the total detection probability for the entire sensor system to recognize the hidden paths. The resulting detection is difficult to determine in sensor systems. The strongest single detection was considered the detection probability of the system. For different targets and environments, different correlation tensors can be applied. This tensor can be determined from measurements. A simple and obvious solution is to use the maximum single detection probability when combining detections of multiple sensors. This case is close to the worst case, which increases the system’s reliability, but there may be negative values in the correlation tensor so that it can be better than the actual worst case. The objective can be written as the minimum observations taken from each route’s maximum observation in Equation (1), where $p_{d}$ is a single detection probability.

(1) $$O_{min}=1-min_{|object}\left(max_{|route}\left(max_{|sensor}\left(p_{d}\right)\right)\right).$$

Applying the objective as a cost function resulted in highly variable costs and frequent population exchange during optimization. The cost function has been modified to improve the quality of the optimization. The number of intruders was increased, and as a standard solution in the low batch sample proportion teachings, the principle of momentum was applied. In Equation (2), the average detection of the objects was calculated. The minimum detection (Equation (1)) and the mean detection (Equation (2)) were weighted ($w_{o}$) in Equation (3). The previously computed values have also been considered in the resulting cost (*C*) in Equation (4). Thus, the momentum principle was realized with the weight factor ($w_{c}$). In Equation (4) *n* denotes the number of simulations. Compared to fixed-structure optimization tasks, it is not practical to consider historical values with greater or uniform weighting. By changing the structure, some sensors can be replaced or combined for a more optimal result despite their excellent performance.

(2) $$O_{mean}=1-mean_{|object}\left(max_{|route}\left(max_{|sensor}\left(p_{d}\right)\right)\right)$$

(3) $$O_{ext}=w_{o}\cdot O_{min}\left(p_{d}\right)+\left(1-w_{o}\right)\cdot \left(O_{mean}\left(p_{d}\right)\right)$$

(4) $$C\left(n\right)=w_{c}\cdot C\left(n-1\right)+\left(1-w_{c}\right)\cdot O_{ext}\left(n\right).$$

A secondary goal of the hierarchical task is to minimize the number of sensors used. Based on the hierarchy, two levels of cost function were applied. The first level of the cost function is to secure the hidden passage, and the second level minimizes the number of sensors. The two-level design was implemented using two cost levels. Any arrangement that can permanently minimize passage will be one step lower in cost, and its goals will be expanded by reducing the number of sensors applied.

### 4.2. Individuals

**Figure 6:**
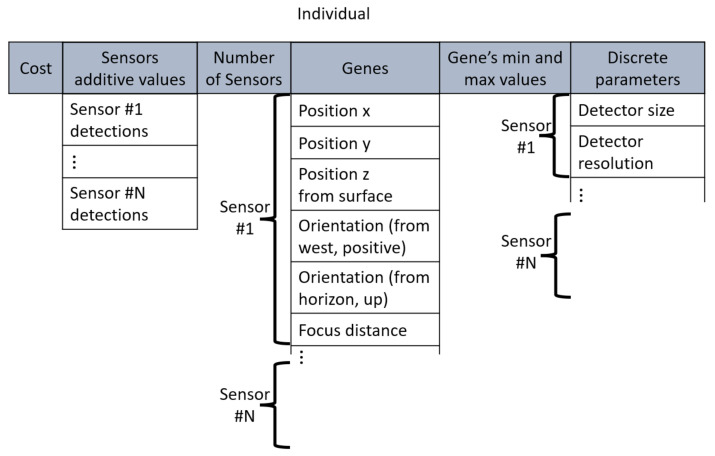
Structure of an individual.

Individuals consisted of varying amounts of sensors. Each sensor had fixed and variable parameters. Fixed parameters were detector size, resolution, and the range of the variable parameters. Variable parameters are position, vertical and horizontal orientation, and focal length. During optimization, the specified parameters could only take discrete values, and the variable parameters were continuous. The structure of the individuals is shown in Figure 6.

### 4.3. Optimization Method

The Bacterial Evolutionary Algorithm (BEA) was used for optimization [62]. The BEA consists of bacterial mutation and gene transfer, shown in Algorithm 2. BEA has been applied to a wide range of problems, for instance, optimizing the fuzzy rule bases [62,63], feature selection [64], data clustering [65], and combinatorial optimization problems [66].
**Algorithm 2** Bacterial Evolutionary Algorithm.**function**Parameter initialization(params)    $N_{pop}\leftarrow$ Number of individuals    criteria← Stop criteria    $opt_{pi}\leftarrow$ Options of population initialization    $opt_{bm}\leftarrow$ Options of bacterial mutation    $opt_{gt}\leftarrow$ Options of gene transfer**function**Population initialization($opt_{pi},N_{pop}$)    **parallel**($N_{pop}$) create and evaluate population**while**criteria**do**    **function** Bacterial mutation($opt_{bm},Population$)    **function** Gene transfer($opt_{gt},Population$)**return**Best individual

**Figure 7:**
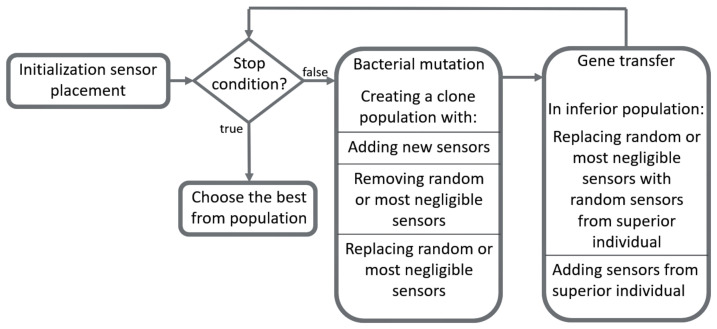
Bacterial Evolutionary Algorithm used for sensor placement.

**Figure 8:**
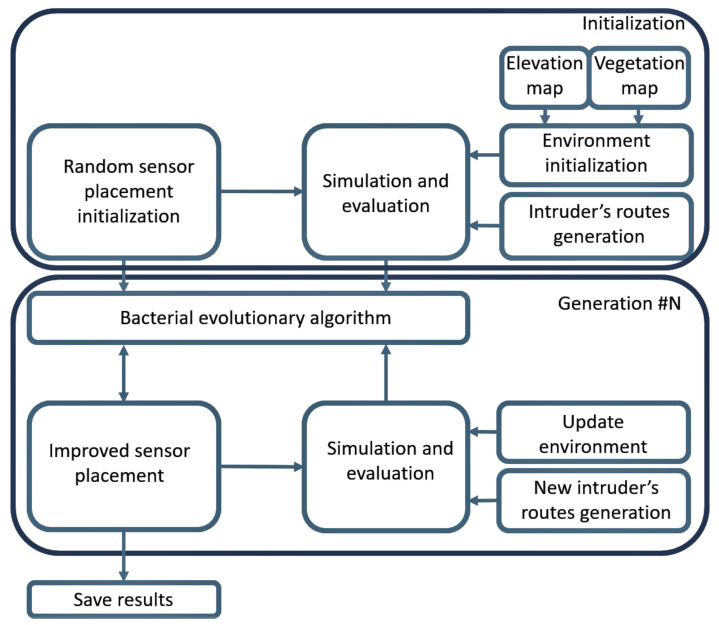
Optimization flow chart.

During bacterial mutation, random sensors or sensors with the lowest added value were chosen. Sensors may have left the individual, new sensors may have joined the individual, or sensors may have been replaced. During gene transfer, one or more sensor from the better-performing individual was transferred to a lower-performing individual or replaced with a sensor from the better individual. The replaced sensors were with the most negligible additive value or selected randomly. The algorithm and the operators used are shown in Figure 7. The optimization process is shown in Figure 8.

A sensor’s additive value is calculated as the sum difference between the maximum detection of each intruder in the whole sensor system and the detection without that sensor. The total additive value of a sensor cannot be calculated due to the infinite possible path and the change of the sensor placement. Still, it can be estimated and summed with the previous values in each simulation.

## 5. Experimental Results

During the experiments, a 1×1×1 km area was simulated. In each iteration, the cloud map has been updated, and new paths have been initialized. 20%–80% division of random sensors or the ones with the estimated lowest added values were modified. The spontaneous mutation has weighed less due to the lack of local search. First, the sensors were selected for clone creation, and then the listed mutations occurred on them, in 30%–30%–40% of cases, a new sensor was added, removed, or replaced. The sensor’s numbers were ranged and considered in selecting the mutation operation. During gene transfer, the chance of sensor transfer and sensor replacement was 50%–50%. A sensors’ additive value was calculated based on a 50%–50% weighting of the previously accumulated and current additive values. Thus, the current value got more significant weight. In general, high-resolution sensors came to the fore during optimization. Several acceptable solutions have emerged for different amounts of sensors. Low-resolution sensors can be helpful in some cases. Due to the mutation operator used, sensors with minimal added value were included temporarily and for more iteration in the case of a larger number of sensors. This error can be fixed by using a local search method. The Bacterial Memetic Algorithm (BMA) [67] complements BEA with local search. This algorithm has several variants with different local search techniques such as the Levenberg–Marquardt algorithm [67], Simulated Annealing [68], Hill climbing, and discrete local search [69,70]. In depicting the results, the sensors’ detection distance was plotted based on the 4-pixel projection of a one-meter target. In general, the sensors had difficulty detecting objects at high altitudes.

**Figure 9:**
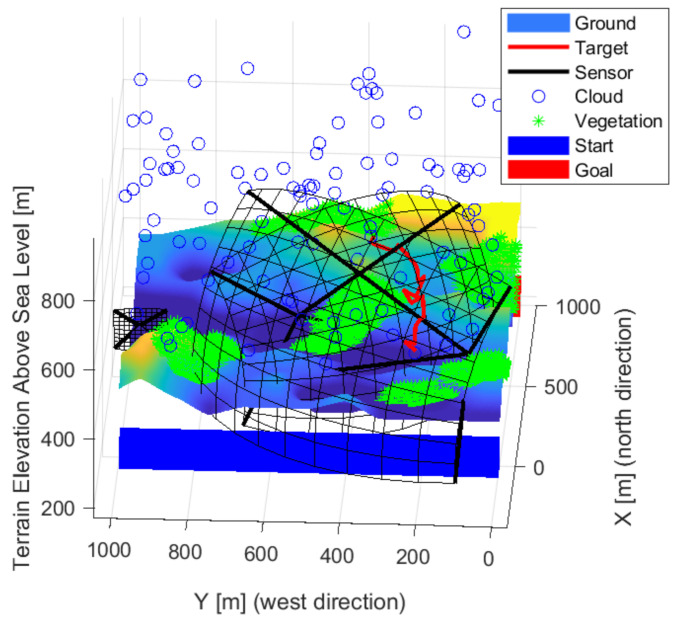
The solution in the case of the three sensors front-wise.

**Figure 10:**
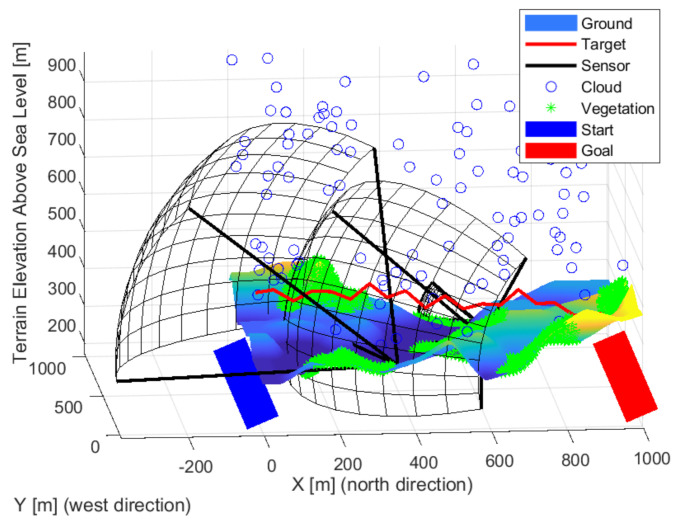
The solution in the case of the three sensors from the side view.

**Figure 11:**
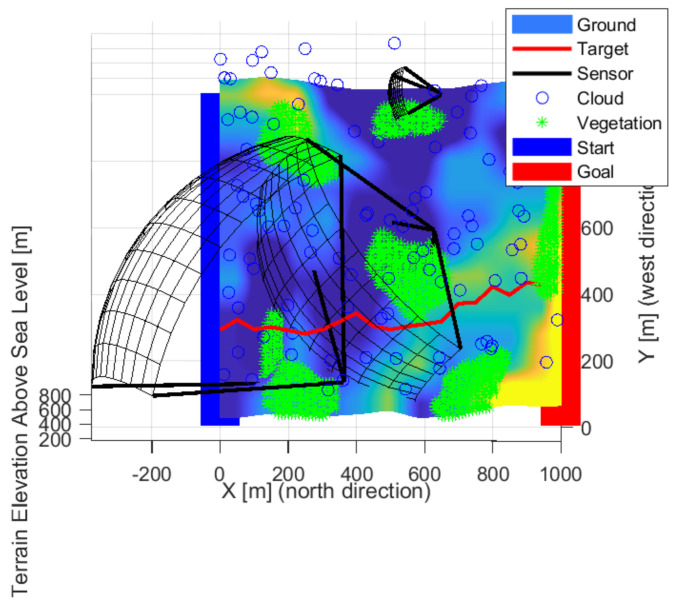
The solution in the case of the three sensors from above.

Figure 9, Figure 10 and Figure 11 show the solution for three sensors. Two sensors facing crossed, the fields of views meet this to cover most of the space. The third sensor is located independently and covers a path at the edge. The layout is able to cover high altitudes with low detection probability. It is not always possible to detect intruders flying low near the ground. Each sensor looks slightly upwards, but not so much that it is greatly affected by the weather. The effects of the sun can degrade detection based on the orientation of the map and the travel direction of the intruder.

**Figure 12:**
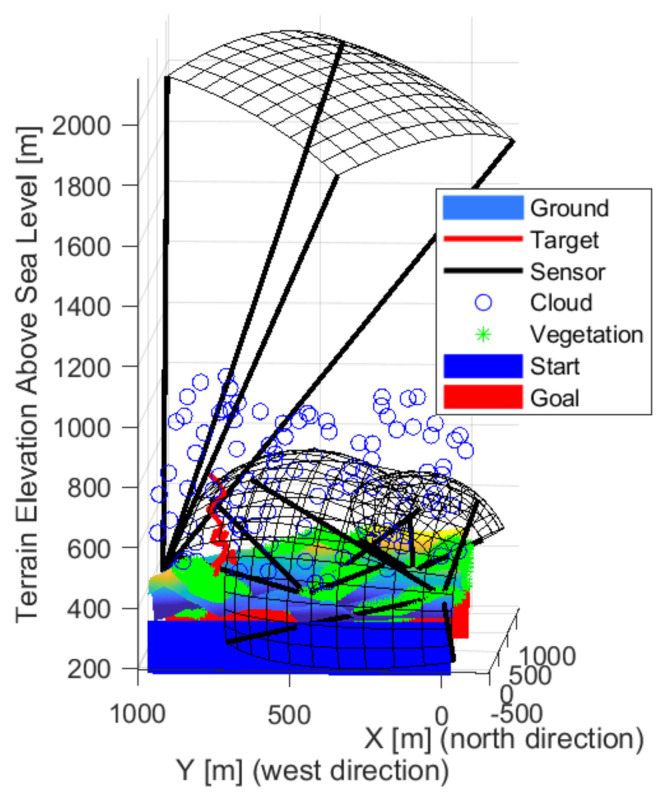
The solution in the case of the four sensors front-wise.

**Figure 13:**
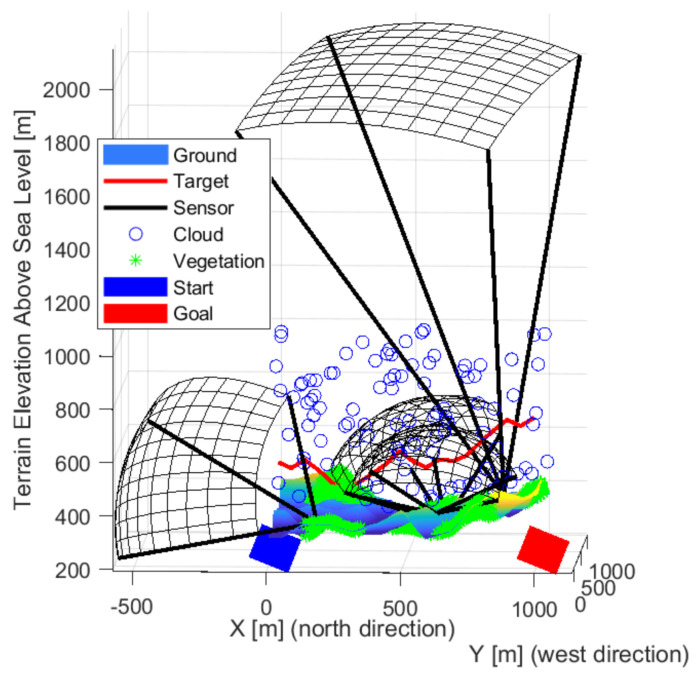
The solution in the case of the four sensors from the side view.

**Figure 14:**
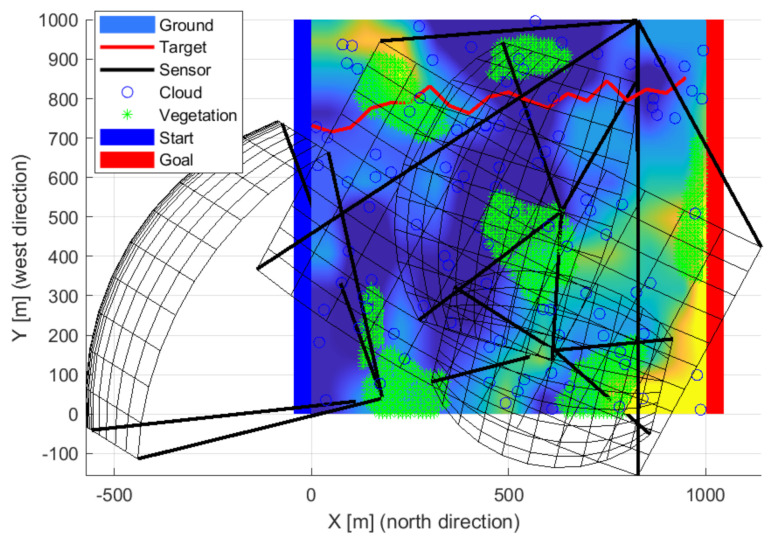
The solution in the case of the four sensors from above.

Figure 12, Figure 13 and Figure 14 show the solution for four sensors. Three sensors were facing up and one forward. The sensors in the valley facing upwards are optimal as the sky background provides better detection. Upward-facing sensors can cover a large area, and the background gives them better detection. Their disadvantage is greater exposure to the weather. The forward-facing sensor is on the edge of the test area, but it covers the edge of the site and as far as possible at the edge of the forest/vegetation to see objects at low altitudes.

Figure 13 shows the sensors looking up and located in the valley are more optimal as the objects are more recognizable in the sky background. The shape of the detection space shows the task’s difficulty, as, from a distance, a sensor can detect less. On the contrary, it sees a smaller area closer. Three sensors look forward and two up. A lower resolution sensor is also included in the middle. Overall, the sensors are better distributed. There are some gaps, but it covers well overall.

**Figure 15:**
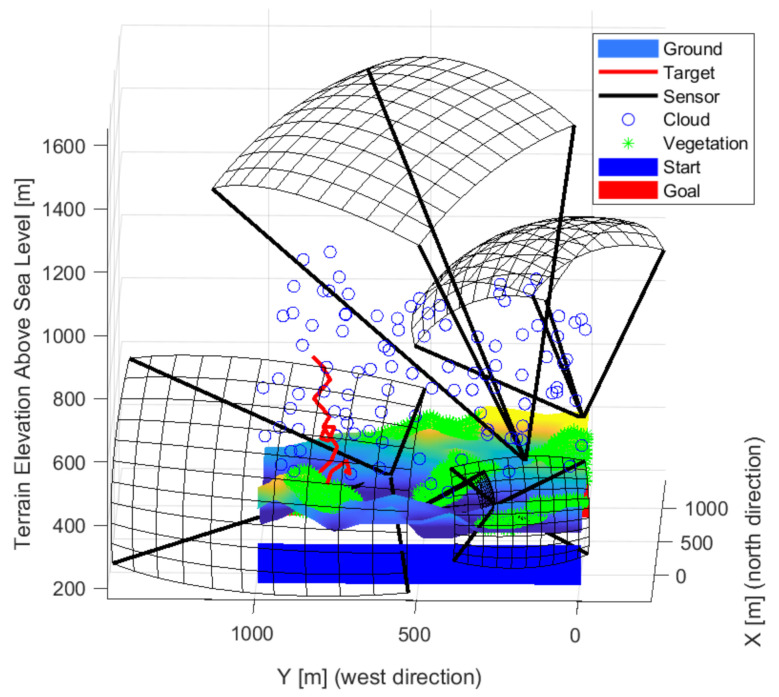
The solution in the case of the five sensors front-wise.

**Figure 16:**
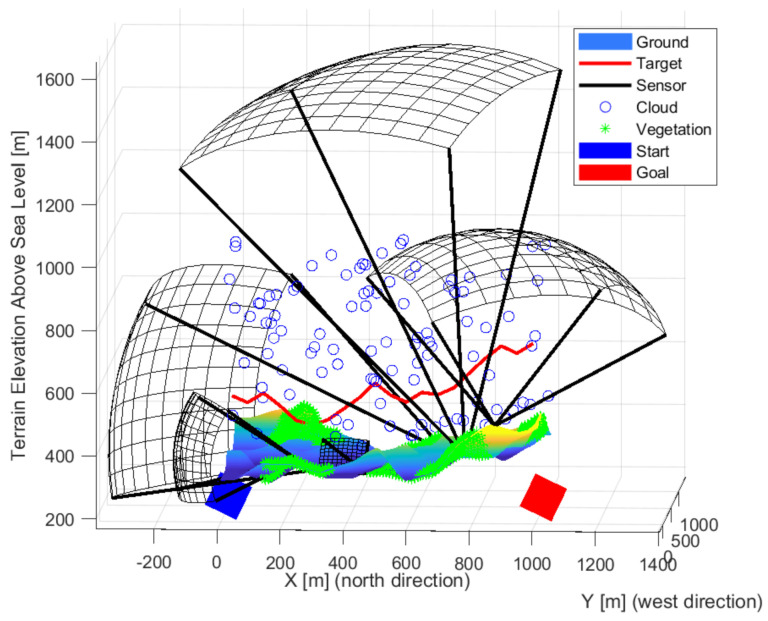
The solution in the case of the five sensors from the side view.

**Figure 17:**
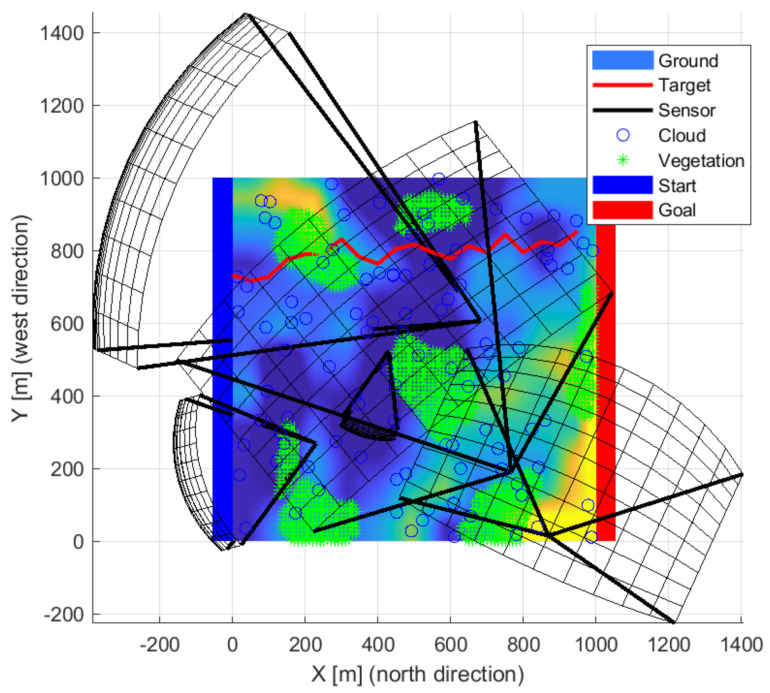
The solution in the case of the five sensors from above.

Figure 15, Figure 16 and Figure 17 show solution for five sensors. They present that we can approach the optimal solution with forward and upward facing sensors.

**Figure 18:**
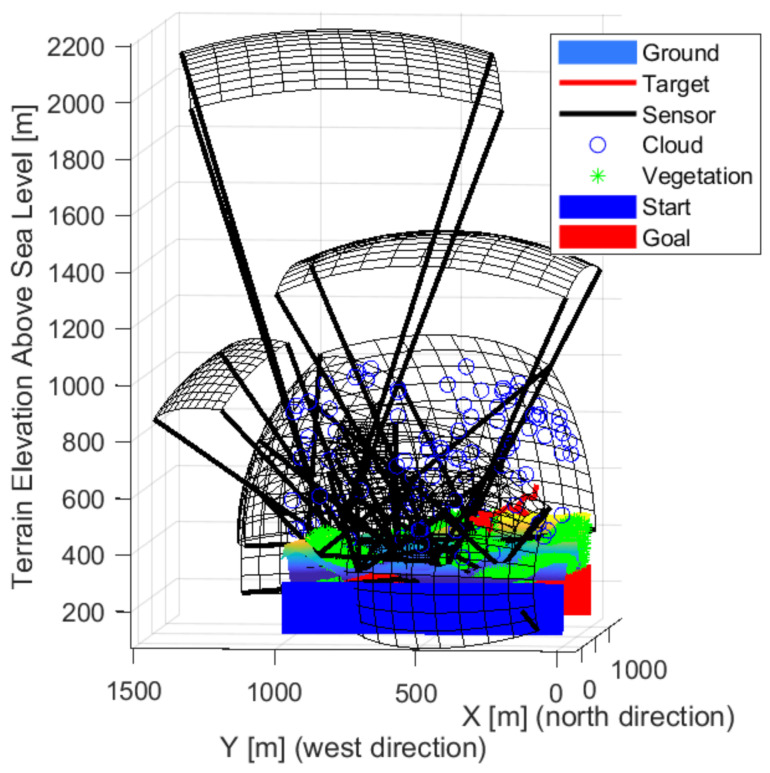
The solution in the case of the thirteen sensors front-wise.

**Figure 19:**
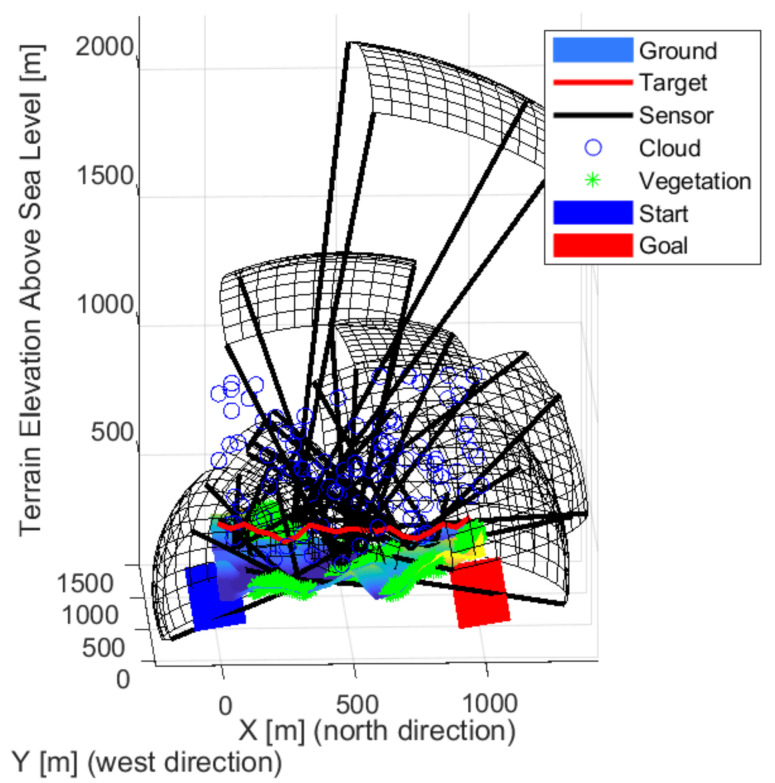
The solution in the case of the thirteen sensors from the side view.

**Figure 20:**
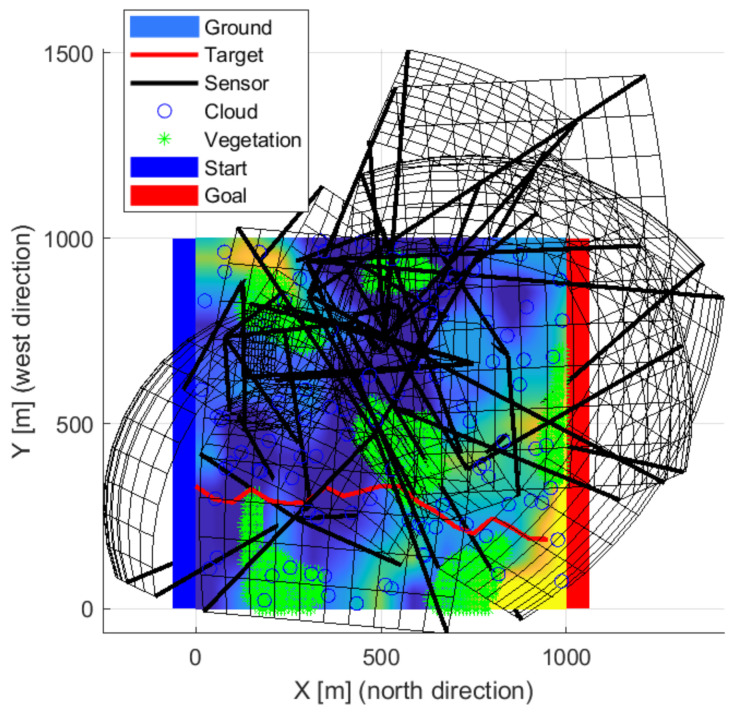
The solution in the case of the thirteen sensors from above.

An arrangement using thirteen sensors is shown in the following Figure 18, Figure 19 and Figure 20, which managed to prevent unnoticed passage. The sensors are located in almost one group, so there is no gap between them. Three upward-sensing sensors are more exposed to the effects of the weather. The visibility ranges of the upward-facing sensors touch each other and cover high-altitude routes well. Lower resolution sensors also appear mainly in valleys.

**Figure 21:**
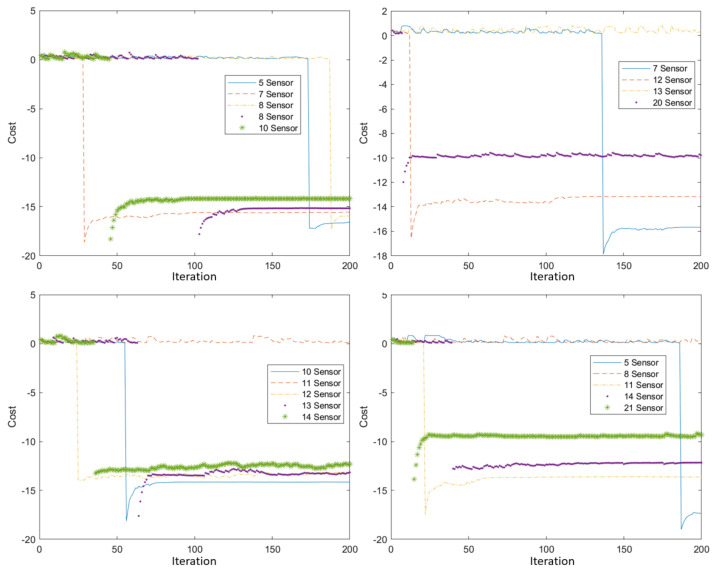
The two-level cost function for different numbers of sensors.

Figure 21 shows the change in cost during optimization for a different number of sensors. Each individual received an initial value of 0.5. The re-initialization of the intruder route has caused a constant fluctuation in costs. Figure 21 shows that the threshold cost is consistently reached in time with different numbers of sensors. Different threshold cost levels have been set for each case. Keeping the threshold cost in two iterations will result in a one-step lower cost per individual and an additional cost per number of sensors. Some individuals in the population also inherit the cost of the original individual through bacterial mutation and gene transfer, and the optimization can run in parallel at the two cost levels. The inheritance of the cost gene is smaller due to the variable number of sensors, and the result of the current simulation is given more weight.

**Figure 22:**
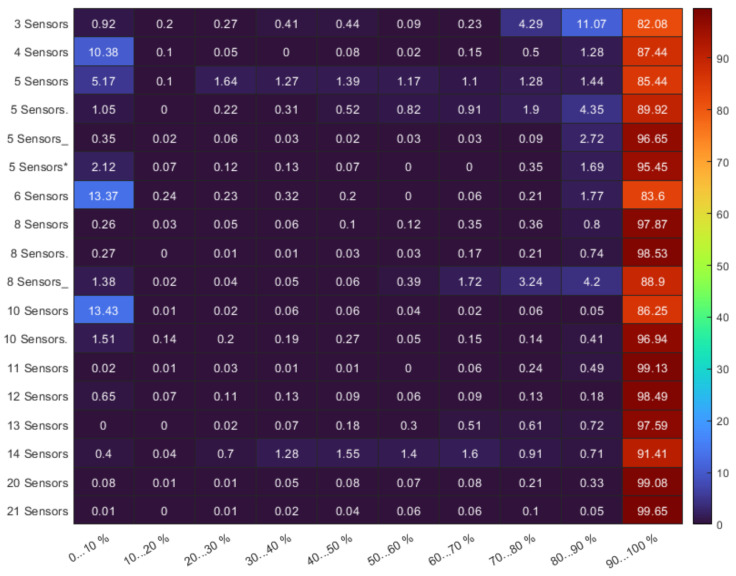
The detection probability distribution for different arrangements and sensor numbers.

Sensor placements were tested with 10,000 random paths. Figure 22 shows an object detection probability distribution for different sensor numbers and layouts. Most detections fall into the high-reliability range of 90…100%. By using intelligent intruders [61], simulations can be made more efficient. There were both superior and inferior solutions for smaller and larger numbers of sensors. At the trend level, it can be seen that more sensors means higher reliability.

## 6. Conclusions and Further Work

We have developed a method based on three-dimensional simulation compared to the currently available quasi-two-dimensional methods to prevent detection-free passage. Instead of the homogeneous or near-homogeneous detection models, we optimized a highly inhomogeneous detection model in a dynamic environment. We have developed a method based on 3D simulation compared to the currently available quasi-two-dimensional methods to prevent detection-free passage. Instead of the homogeneous/near-homogeneous detection models, we optimized a highly inhomogeneous detection model in a dynamic environment. We tested a stepped cost function for hierarchical multi-purpose optimization. The applied bacterial evolutionary algorithm was able to optimize the sensor placement. The sensors were positioned correctly in a complex environment even without local operators. For random routes, the majority of intruders (>80%) were detected with a high probability (>90% detection). Due to the simple path generation of intruders, it is more important to investigate ’quasi’ intelligent intruders in the low detection categories. With the presented optimization, we succeeded in preventing undetected intrusions for 10,000 trials in the studied environment. Above the use of five sensors, undetected intrusions (<10% detection) and uncertain detections (<50% detection) can be reduced to less than 1% for intruders at low (>0.5 m) and high (<700 m) altitudes. In addition to flying intruders, the case of standing humans is also included in the parameter range. By using ten sensors, undetected intrusions and uncertain detections can be reduced to <0.1%. In addition to detection, classification is another crucial aspect. Classification is only possible with a high detection probability (>70%). The results show that a high percentage (>80%) of the intruders has a high probability of detection (>90%) could be classified with reasonable confidence. A high percentage (99%) of the intruders are expected to classify using more than twenty sensors with an acceptable probability.

Further research aims to extract environmental data based on image segmentation automatically. Other goals are to improve the environment model with the effects of weather and sun, apply intelligent intruders, and implement appropriate local search. The implemented simulation is suitable for extracting gradient approximation. Due to discrete simulations, a local search procedure using momentum would be the most efficient [71].

## Figures and Tables

**Table 1:** Background impacts on the signal.

Environmental Element	Sign Decrease [%]
Clear sky	0
Clouds	20 · cloud’s density
Ground	[0…50] predefined
Walls	[0…50] predefined
Vegetation	50 · vegetation’s density
